# Raising Money for Hospitals

**Published:** 1923-02

**Authors:** G. Russell Chapman

**Affiliations:** 22a, Regent Street, W.1


Sir,—I have rarely seen an article on this important subject with which I find myself in such cordial agreement as with that published in your December issue. Read in conjunction with the related paragraphs of your leader, it constitutes a valuable contribution to the burning subject that is causing Hospital Boards such present anxiety.

The inadequacy of the financial resources of most voluntary hospitals is mainly due to two root causes—the lack or insuffiiency of endowments and a scarcity of regular annual ccontributions. It has become the custom during recent years to allow annual deficiencies to accumulate and then announce a crisis, issue an S.OS. appeal, and make an exhausting special effort. to wipe out arrears and re-establish financial equilibrium. Recurrent efforts of this description have weakened their force, and during the past few years they have shown, in general, decreasing results. Where new capital expenditure is involved the special effort has a greater justification, and where the sympathies and energies of friends and supporters have not been exhausted in making special efforts for maintenance funds, an intensive appeal for the capital sum required is undoubtedly the best method of securing a large sum of money.

Every special effort, however, tends to reduce regular annual income, whether the campaign be for debt, maintenance or new capital expenditure. This is clearly shown by the examination of a large number of hospital reports ; and in assessing the value of special appeals the amount of the reduction in annual subscriptions and donations received during the same year must be taken into account. No single reform or improvement can be said to constitute the key to the present situation, but a number of reforms might with benefit be considered by hospital managements, of which the following are the principal :—

1. The placing of the finance appeal organisation of the hospital under the direction or supervision of an expert, whose services should be retained in an advisory and consultative capacity, the hospital paying and controlling their own appeal staff, whose work, however, would be subject to the scrutiny of the finance adviser. Such action would ensure the adoption of sound appeal methods, and the elimination of wasteful and irritating activities together with the overlapping and unscientific efforts which, without producing adequate results, exhaust the energies of helpers, try the patience of supporters, and lead to exasperation, worry, and finally criticism of the hospital itself.

2. The concentration upon building up of an adequate endowment fund, providing through gifts and legacies a substantial regular income from investments and securities, the securing of a large number of new annual contributions, together with the increasing of the amounts contributed by present supporters whose gifts have not been raised proportionately with the increased cost of hospital administration and the lowered purchasing power of sterling.

3. The institution of what has come to be called a "Contributory Scheme," whereby employés pay a fixed regular contribution, or a percentage contribution on wages received, ensuring their free treatment at the hospital in the event of injury or disease, and at the same time providing a regular source of income.

4. The inadvisability of S.O.S. appeals, except in the greatest emergency, as tending to produce a reaction and create the feeling that in no circumstances can voluntary hospitals be made self-supporting, and that efforts to this end will merely postpone and not obviate the taking over or subsidising of these institutions by the State.

5. Where an emergency exists and a special appeal becomes necessary, it should be planned and carried out under expert advice, restricted to a given period, and organised so as to disturb as little as possible the normal and regular collection of the hospital's income.

Sources, in the case of practically every hospital in the land, exist from which an increased annual revenue can be obtained, and methods for securing this potential income have been devised and operated successfully. The conditions and circumstances of hospitals vary greatly, and it is always necessary to consider the financial problem of each in conjunction with such local circumstances. This, however, should be done under expert advice, and where business men are represented on Hospital Boards it will readily be conceded that amateur and inexperienced methods, or the individual and uncoordinated efforts of well-meaning but unskilled helpers, will not make for success in hospital finance appeals any more than such methods will attain success in business affairs.

Faced with the accumulating deficits of the lean financial years following the war, the position accentuated by contrast with the previous years of plenty, Hospitals Boards and their Superintendents have been driven to reorganisation schemes and economies that in the long run will undoubtedly prove beneficial; but they have inevitably thrown additional burdens upon official shoulders. Until Hospital Boards have relieved their Secretaries and Superintendents, whose whole time is now required for securing efficiency and economy of administration, from the burden of finance appeal work, and placed this department under the control or guidance of experienced hands, it could not truthfully be said that the voluntary system had been adequately tested, much less failed.

G. Russell Chapman.

22a, Regent Street, W.1.

